# The relationship between ageism, loneliness, and anxiety in widowed older adults: cognitive function as a moderator in the mediated model

**DOI:** 10.3389/fpsyg.2025.1624197

**Published:** 2025-06-18

**Authors:** Haohui Shen, Guoliang Pan, Mengge Zhang, Xiuwen He, Mingyang Yao, Yilong Yang

**Affiliations:** ^1^Department of Health Policy and Management, School of Public Administration, Hangzhou Normal University, Hangzhou, China; ^2^Shenyang Mental Health Center, Shenyang, China

**Keywords:** anxiety, ageism, loneliness, cognitive function, widowed older adults, moderated mediation model

## Abstract

**Objective:**

As the global population ages and traditional family support structures decline, mental health issues—particularly anxiety—among widowed older adults have become increasingly prevalent. Studies have shown a positive correlation between ageism and anxiety disorders in older bereaved individuals, but the underlying mechanism by which ageism affects anxiety in this population has not yet been clarified. This study aimed to examine whether loneliness mediates the relationship between age discrimination and anxiety disorders, and whether cognitive functioning moderates this mediation.

**Methods:**

A random sampling approach was used to select 406 older adults who have lost their spouse from Shenyang, China. The Generalized Anxiety Scale (GAD-7), Perceived Age Discrimination Scale (PAD), UCLA Loneliness Scale (UCLA-LS), and Alzheimer’s Disease 8-Item Screening Instrument (AD-8) were used to assess the status of anxiety, ageism, loneliness, and cognitive functioning, respectively. Moderated mediation models were analyzed by SPSS PROCESS version 4.0 software.

**Results:**

Age discrimination had a significant direct effect on anxiety in widowed individuals in later life. Loneliness partially mediated the effect of age discrimination knowledge on anxiety. In addition, the second half of the path of the indirect effect was moderated by cognitive functioning. The indirect effect of loneliness on anxiety was enhanced when cognitive functioning was poor.

**Conclusion:**

Loneliness enhances the positive association between ageism and anxiety in widowed older adults, in which cognitive functioning plays a moderating role. These findings suggest the need for targeted psychosocial interventions that prioritize cognitive health and social engagement to reduce anxiety among bereaved older adults.

## 1 Introduction

Currently, the global aging population is steadily increasing. Specifically, data from the 7th Census indicate that by 2020, individuals aged 65 and older constituted 13.5% of the population, representing nearly a 5 percentage points rise since 2010 (National Bureau of Statistics of China [NBS], 2021). At the same time, the growing elderly population is closely linked to heightened mental health concerns. According to statistics, approximately 14% of adults over 60 worldwide suffer from mental illnesses (World Health Organization [WHO], 2023). Notably, depression and anxiety are among the most common and impactful mental health issues (World Health Organization [WHO], 2020). Widowed older adults, a particularly vulnerable subgroup, experience higher levels of stress and are more susceptible to physical and mental health issues due to the loss of emotional and financial support following spousal bereavement (Ansari et al., 2021; Srivastava et al., 2021). Moreover, Singham et al. (2021) found that widowhood accelerates cognitive decline in older adults over time. Similarly, a study on Chinese widowed individuals reported significantly higher generalized anxiety disorder (GAD) scores among widowed adults compared to their non-widowed peers, with notable co-morbidity with depression (Xue et al., 2024). A recent systematic review and meta-analysis reported that approximately 28% of older adults worldwide experience anxiety symptoms, underscoring the substantial mental health burden in this population (Shafiee et al., 2025). Anxiety not only causes physical harm but also exacerbates other psychological conditions. Studies have found that anxiety is an independent risk factor for kidney stone disease (Gao et al., 2024). Additionally, women with depression or anxiety tend to accumulate chronic conditions at a faster rate (Bobo et al., 2022). Moreover, they frequently exhibit heightened levels of depression and anxiety symptoms. Therefore, further research is essential to explore the factors influencing anxiety and the mechanisms through which they alleviate GAD symptoms in older adults, especially widowed individuals.

Ageism is defined as the stereotyping, prejudice, and discrimination directed at others or oneself due to age. Globally, ageism against older adults is highly prevalent, with at least one in two individuals holding ageist attitudes toward this population. In the United States, the economic burden of healthcare costs attributed to ageism has reached $63 billion (Levy et al., 2020). Multiple cross-sectional studies have demonstrated that individuals perceiving greater levels of ageism tend to exhibit higher depression and anxiety symptoms. Moreover, elevated anxiety levels are strongly associated with poorer mental health scores (Carden et al., 2022; Allen et al., 2022). While existing research has clearly established the link between ageism and anxiety, most studies have focused on older adults in general, with limited attention to widowed older adults as a distinct group. Furthermore, the pathways through which ageism influences anxiety remain insufficiently explored, highlighting the need for further studies aimed at preventing and alleviating anxiety symptoms in this vulnerable population.

Loneliness arises when there is a perceived gap between the social connections an individual desires and those they actually experience (Akhter-Khan et al., 2023). Moreover, ageism is frequently associated with elevated levels of loneliness. According to the multi-maneuver model of interpersonal rejection, including discrimination and exclusion, proposed by Smart Richman and Leary (2009), individuals encountering such situations often adopt withdrawal or avoidance behaviors, eventually leading to social isolation. Meanwhile, research has shown that all indicators of social isolation are linked to increased loneliness in older adults (Taylor, 2020). Additionally, a significant positive correlation between anxiety and loneliness has been consistently demonstrated (Müller et al., 2025). On the other hand, a study on factors predicting loneliness among Chinese older adults revealed that widowhood significantly increases loneliness across both gender and age groups (Jiao et al., 2024). Consequently, loneliness may serve as a mediator in the relationship between ageism and anxiety among widowed older adults.

Cognitive function has emerged as a critical factor influencing health and quality of life among middle-aged and older adults. According to emotion regulation theory, cognitive impairment may reduce an individual’s capacity to reappraise or suppress negative emotions (Mcrae, 2016). Beyond influencing emotions and attitudes, stressful events such as ageism may interact with cognitive function, indirectly affecting mental health (Ahn et al., 2024). This interaction is particularly relevant for widowed older adults, who are more vulnerable to cognitive impairment (Singham et al., 2021). Furthermore, in older adults, reduced cognitive functioning is associated with higher levels of anxiety (Gulpers et al., 2022). So we suggest that cognitive impairment may amplify the effects of stressful events, such as ageism, on anxiety. Recent studies have explored the moderating role of cognitive function in the relationship between social isolation and anxiety (Hwang et al., 2024), as well as its influence on the link between loneliness and social resources (Burholt et al., 2017). Based on emotion regulation theory and existing research, we propose that cognitive function moderates the relationship between ageism and anxiety disorders in widowed older adults. Thus, the hypotheses of this study are:

*H1*. Among widowed older adults, exposure to ageism is positively related to anxiety symptoms.

*H2*. The effect of ageism on anxiety among bereaved elderly is mediated by feelings of loneliness.

*H3*. Cognitive function acts as a moderator within the aforementioned mediation model.

This study contributes to the literature by examining loneliness as a mediator and cognitive function as a moderator in the relationship between ageism and anxiety, providing new insights into the psychological mechanisms and boundary conditions of ageism’s impact on mental health.

## 2 Methods

### 2.1 Study design and participant

In this study, Shenyang City, located in Liaoning Province, served as the primary sampling unit. A total of 30 units were selected based on regional divisions and economic development, including 13 county-level administrative districts, one county-level city, and two counties. Eligible participants were required to meet the following criteria: (1) be aged 60 years or older; (2) have a marital status of widowhood at the time of the survey; and (3) possess the ability to read and comprehend the survey materials. Upon obtaining written informed consent from all participants, the research team conducted centralized data collection at community activity centers within each selected survey unit. The survey was administered in a paper-based, self-administered format, with participants independently completing the questionnaire on site. Although the questionnaires were self-administered, all field researchers had undergone standardized training and remained present throughout the process to provide uniform instructions and offer non-intrusive clarification when participants encountered difficulties in understanding specific items. The average time required to complete the questionnaire was approximately 20 min per participant. A total of 475 completed questionnaires were collected. Following data screening and organization, 406 valid questionnaires were retained for analysis. This study was approved by the Shenyang Mental Health Center, Liaoning Province, China (Approval No. 2024004), and was conducted in strict accordance with ethical standards throughout the research process.

### 2.2 Measures

#### 2.2.1 Sociodemographic characteristics

Demographic characteristics included gender, age, educational attainment (categorized as 0–6 years, 7–14 years, or 15–21 years), religious affiliation (categorized as “have” or “none”), relationship with friends (rated as very bad, not very good, average, fairly good, or very good), and life satisfaction (rated as very dissatisfied, dissatisfied, neutral, satisfied, or very satisfied).

#### 2.2.2 Anxiety

This study employed the 7-item Generalized Anxiety Disorder Scale (GAD-7) to evaluate anxiety levels among older adults (Spitzer et al., 2006). The scale comprises 7 items, each rated on a 4-point Likert scale ranging from 1 (“not at all”) to 4 (“almost every day”). The total score ranges from 7 to 28, with higher scores reflecting greater levels of anxiety. In this study, the GAD-7 demonstrated excellent internal consistency, with a Cronbach’s alpha coefficient of 0.928.

#### 2.2.3 Ageism

The Perceived Age Discrimination Scale, developed by Vauclair et al. (2016), was used to assess widowed older adults’ subjective experiences of ageism. The scale consists of three items: “In the past year, have you felt that someone showed prejudice or mistreated you because of your age?,” “In the past year, have you felt disrespected, such as being ignored or looked down upon, because of your age?,” and “In the past year, has anyone mistreated you because of your age, such as insulting, abusing, or denying you services?.” Responses were recorded on a 5-point Likert scale ranging from 0 (“never”) to 4 (“very often”), with total scores ranging from 0 to 12. Higher scores indicate greater perceived age discrimination. This three-item measure was originally included in Round 4 of the European Social Survey (ESS) and has demonstrated measurement invariance across age groups and countries, supporting its cross-cultural validity (Bratt et al., 2018). In the present study, the scale showed excellent internal consistency, with a Cronbach’s alpha coefficient of 0.930.

#### 2.2.4 Loneliness

This study utilized the UCLA Loneliness Scale (UCLA-LS) to measure levels of loneliness (Russell, 1996). The scale comprises 20 items, rated on a 4-point Likert scale ranging from 1 (“never”) to 4 (“always”), with 9 items reverse-scored. The total score ranges from 20 to 80, with higher scores reflecting greater loneliness. In this study, the UCLA-LS demonstrated good internal consistency, with a Cronbach’s alpha coefficient of 0.879.

#### 2.2.5 Cognitive function

The AD8 scale is a tool designed to assess cognitive abilities in older adults, addressing common cognitive issues encountered in daily life, such as memory, attention, and judgment (Galvin et al., 2005). The scale comprises 8 items, each rated on a 2-point Likert-type scale, ranging from 0 (“no”) to 1 (“yes”). The total score ranges from 0 to 8, with higher scores reflecting greater levels of cognitive impairment. In accordance with standard guidelines, a cut-off score of ≥2 was used to indicate potential cognitive impairment. In this study, the Cronbach alpha coefficient for AD8 was 0.813.

### 2.3 Statistical analysis

Descriptive analyses were performed to examine socio-demographic characteristics, and Pearson correlation analyses were used to explore the relationships among variables, including ageism, loneliness, anxiety disorders, and cognitive function. Mediation and moderated mediation effect analyses were conducted using the PROCESS macro program in SPSS (Hayes, 2017). Bias-corrected 95% confidence intervals (CIs) were estimated based on 5,000 bootstrap samples.

In this study, Model 4 of the PROCESS macro was used to test whether loneliness mediated the relationship between ageism and anxiety disorders. A significant mediating effect was determined if the 95% confidence interval for the indirect effect (path a*b) did not include zero.

**Figure 1:**
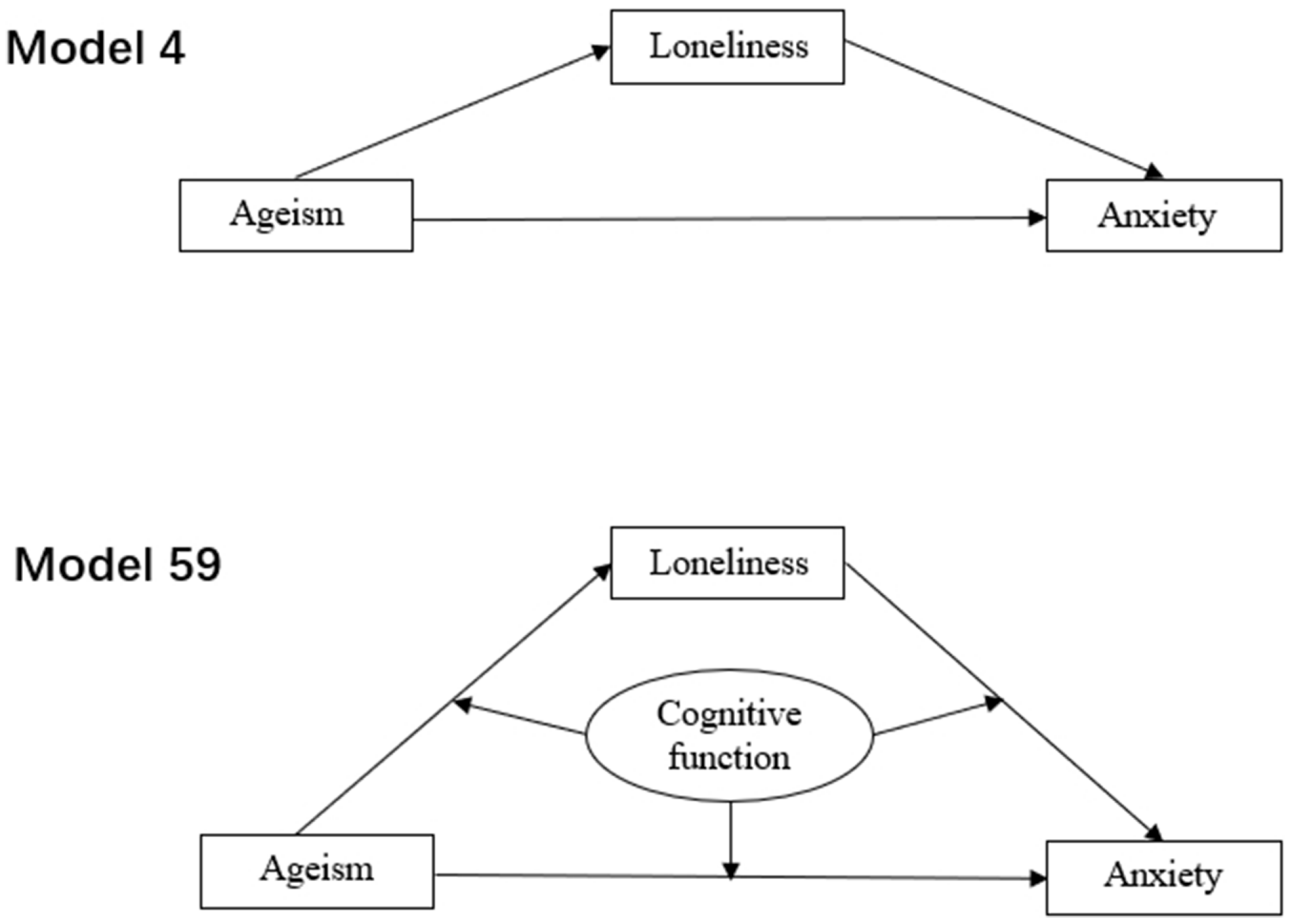
Hypothesized mediation and moderated mediation models illustrating the proposed relationships. Model 4 (top panel) presents a hypothesized mediation model in which loneliness may mediate the relationship between ageism and anxiety. Model 59 (bottom panel) assumes that cognitive function may moderate the paths within the above mediation model, forming a moderated mediation structure.

Next, Model 59 was employed to examine the moderating mediation effect, testing whether cognitive function moderated both the direct and indirect effects of ageism on anxiety disorders. Similarly, a significant moderating mediation effect was identified if the 95% confidence interval for the interaction effect did not include zero. The path diagrams for the two models are shown in Figure 1. Additionally, simple slope analyses were performed to further investigate the moderating effects, and the results have been included in the Supplementary material.

## 3 Results

### 3.1 Demographic characteristics

Table 1 presents the demographic characteristics of the study population. Among the 406 participants, 42 (10.3%) were male. The age distribution included 183 (45.1%) aged 55–70 years, 196 (48.3%) aged 71–85 years, and 27 (6.6%) aged 86–99 years. A comparative analysis of anxiety levels based on demographic characteristics revealed no statistically significant differences in anxiety scores across gender, age, education, or religion. Older adults who reported better relationships with friends exhibited lower anxiety scores. Additionally, older adults with higher life satisfaction reported lower anxiety scores compared to those with lower satisfaction levels. The normality of the main continuous variables, including ageism, loneliness, anxiety, and cognitive function scores, was evaluated using the Shapiro–Wilk test and visual inspection of Q–Q plots. In addition, we conducted multicollinearity diagnostics using Variance Inflation Factors (VIF), and no significant multicollinearity was detected among the variables. The results indicated that all variables exhibited approximately normal distributions, supporting the appropriateness of using parametric statistical methods in subsequent analyses.

**Table 1:** Social-demographic characteristics and comparison of anxiety scores in different groups.

Variable	Number	Anxiety (*M* ± SD)	*F*	*P*
Gender			0.01	0.95
Male	42	8.60 ± 3.38		
Female	364	8.65 ± 3.33		
Age			2.47	0.09
55–70	183	8.74 ± 3.40		
71–85	196	8.38 ± 2.85		
86–99	27	9.85 ± 5.40		
Education attainment			0.25	0.78
0–6	159	8.54 ± 3.08		
7–14	223	8.74 ± 3.60		
15–21	24	8.38 ± 2.34		
Religious belief			0.55	0.46
Have	56	8.91 ± 3.60		
None	350	8.60 ± 3.30		
Relationship with friends			7.98	<0.001^***^
Very bad	10	8.80 ± 3.36		
Not very good	5	12.40 ± 6.07		
Average	52	10.63 ± 4.41		
Fairly good	161	8.44 ± 3.06		
Very good	178	8.12 ± 2.84		
Life satisfaction			11.8	<0.001^***^
Very dissatisfied	16	7.69 ± 1.78		
Dissatisfied	14	11.21 ± 4.68		
Neutral	48	11.19 ± 5.28		
Satisfied	132	8.73 ± 3.16		
Very satisfied	196	7.85 ± 2.27		

***p < 0.001.

### 3.2 Correlations among the main variables

This study examined the correlations among four variables: anxiety, ageism, loneliness, and cognitive function. The results of the correlation analysis are presented in Table 2.

**Table 2:** Correlation coefficient of ageism, loneliness, anxiety and cognitive function.

Variables	*M*	SD	1. Ageism	2. Loneliness	3. Anxiety	4. Cognitive function
1. Ageism	1.03	1.75	1			
2. Loneliness	36.96	10.55	0.363^***^	1		
3. Anxiety	8.64	3.33	0.323^***^	0.309^***^	1	
4. Cognitive function	1.43	2.21	0.191^***^	0.140^***^	0.456^***^	1

***p < 0.001.

### 3.3 Mediation model

**Figure 2:**
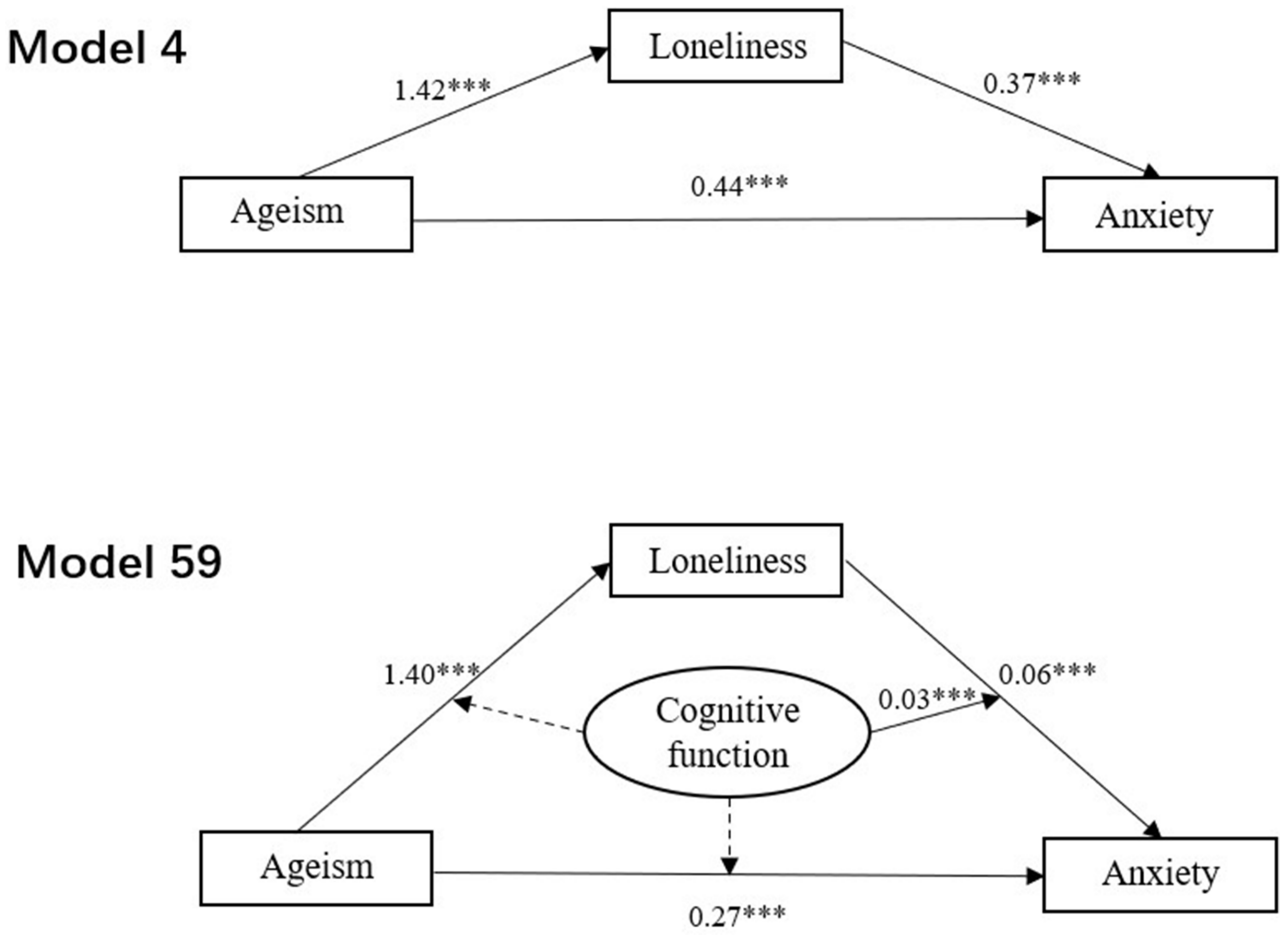
The relevant path coefficients for the mediator model and the final moderated mediator model. *p < 0.05 (two-tailed), **p < 0.01 (two-tailed), ***p < 0.001 (two-tailed).

After controlling for relationships with friends and life satisfaction, ageism remained positively associated with anxiety (*β* = 0.44, *p* < 0.001). When loneliness was included as a mediating variable, both ageism (*β* = 0.37, *p* < 0.001) and loneliness (*β* = 0.05, *p* < 0.01) showed significant positive associations with anxiety. Furthermore, ageism was identified as a significant positive predictor of loneliness (*β* = 1.42, *p* < 0.001). The path coefficients illustrating the relationships among ageism, loneliness, and anxiety are presented in Figure 2.

As shown in Tables 3, 4, the 95% bootstrap confidence intervals for the mediating effect of ageism on anxiety through loneliness did not include 0. This finding indicates that ageism not only had a direct effect on anxiety but also indirectly influenced anxiety via loneliness. The direct effect was 0.37 (95% CI: 0.22, 0.57), and the mediating effect was 0.07 (95% CI: 0.03, 0.13). These effects accounted for 84.1 and 15.9% of the total effect (0.44; 95% CI: 0.30, 0.58), respectively.

**Table 3:** A regression analysis of the mediating role of loneliness in the relationship between ageism and anxiety.

Variable	Anxiety			Loneliness			Anxiety		
	*β*	SE	*t*	*β*	SE	*t*	*β*	SE	*T*
Ageism	0.44	0.07	6.22^***^	1.42	0.20	6.95^***^	0.37	0.07	4.94^***^
Loneliness							0.05	0.17	3.07^**^
*R* ^2^	0.37			0.54			0.40		
*F*	21.81			53.92			19.0		

***p < 0.001, **p < 0.01.

Relationship with friends and Life satisfaction were analyzed as control variable.

Examining the simple mediation model of Loneliness by Model 4 in PROCESS.

**Table 4:** Analysis of the mediating effect of loneliness between ageism and anxiety.

Effect type	Effect	SE	Boot LLCI	Boot ULCI	*T*
Total effect	0.44	0.07	0.30	0.58	6.22^***^
Direct effect	0.37	0.07	0.22	0.57	4.93^***^
Indirect effect	0.07	0.02	0.03	0.13	

***p < 0.001. SE, standard error; Boot, bootstrapping; LLCL, lower limit of the 95% confidence interval; ULCL, upper limit of the 95% confidence interval.

### 3.4 Moderated mediation model

Table 5 presents the results of the moderated mediation analysis. Loneliness (*β* = 0.06, *p* < 0.001) and cognitive function (*β* = −0.81, *p* < 0.01) were all significantly negatively associated with anxiety. The interaction between loneliness and cognitive function (*β* = 0.03, *p* < 0.001) on anxiety was found to be statistically significant. The results indicated that cognitive function did not moderate the direct effect in the hypothesized model. It moderated the second segment of the indirect path, specifically the relationship between loneliness and anxiety symptoms. The final moderated mediation model and its results are displayed in Figure 2. This finding suggests that cognitive function positively moderated the association between loneliness and anxiety symptoms among widowed older adults.

**Table 5:** The moderated mediation effect in the relation between ageism and anxiety.

Variable	(Outcome variable: loneliness)			(Outcome variable: anxiety)		
	*β*	SE	*t*	*β*	SE	*t*
Ageism	1.40	0.21	6.77^***^	0.27	0.07	4.10^***^
Loneliness				0.06	0.02	3.61^***^
Cognitive function	0.30	0.36	0.84	−0.81	0.32	−2.49^*^
Ageism*Cognitive function	−0.05	0.11	−0.44	0.01	0.03	0.38
Loneliness*Cognitive function				0.03	0.01	4.49^***^
*R* ^2^	0.54			0.59		
*F*	32.4			30.4		

***p < 0.001, *p < 0.05.

Relationship with friends and Life satisfaction were analyzed as control variable.

Examining the moderated mediation model of Loneliness and Cognitive function by Model 59 in PROCESS.

To further investigate the moderating role of cognitive function within the mediation model, simple slope analyses were conducted. These analyses involved adding and subtracting one standard deviation from the mean cognitive function score to define high and low cognitive function groups. Table 6 illustrates the moderating effects of varying levels of cognitive function on the relationship between loneliness and anxiety. For a more intuitive presentation of the interaction effect, a simple slopes plot is provided in the Supplementary material. The results indicated that loneliness was a weaker predictor of anxiety in widowed older adults with moderate cognitive impairment compared to those with high cognitive impairment. However, no significant effect was observed in the subgroup with high cognitive function scores. These findings suggest that the predictive effect of loneliness on anxiety intensifies as cognitive function declines in widowed older adults.

**Table 6:** The moderating effect of different levels of cognitive function between loneliness and anxiety.

Cognitive function	Effect size	SE	Boot LLCI	Boot ULCI
M-SD	0.02	0.02	−0.01	0.07
M	0.08^***^	0.02	0.04	0.13
M + SD	0.16^***^	0.05	0.07	0.26

***p < 0.001. SE, standard error; Boot, bootstrapping; LLCL, lower limit of the 95% confidence interval; ULCL, upper limit of the 95% confidence interval.

## 4 Discussion

In this study, we examined the relationship between perceived ageism and generalized anxiety disorder (GAD) in a sample of widowed older adults in China, as well as the potential mechanisms underlying this relationship. The findings indicated that ageism, loneliness, and cognitive function were significantly associated with anxiety disorders, aligning with previous research (Carden et al., 2022; Müller et al., 2025; Gulpers et al., 2022). Specifically, ageism exerted a significant direct effect on anxiety disorders, while loneliness partially mediated this relationship. Higher levels of perceived ageism and loneliness were associated with increased anxiety disorders among widowed older adults. Cognitive function, as a hypothesized moderating variable, moderated the latter segment of the mediation model. Loneliness was a significantly stronger predictor of anxiety disorders among widowed older adults with higher levels of cognitive impairment.

Numerous studies have confirmed a negative relationship between ageism and mental health in older adults (Kang and Kim, 2022); a finding also observed among widowed older adults in this study. Individuals experiencing higher levels of perceived ageism often report greater loneliness. Older individuals who experience age-based discrimination often adopt avoidance behaviors and gradually reduce social participation (Ayalon and Tesch-Römer, 2018), leading to a shrinking social network. Reduced social networks are also associated with increased loneliness (Sun et al., 2024). On the other hand, according to stereotype theory (Barber, 2017), individuals who perceive negative external evaluations are likely to internalize them, forming negative self-perceptions. For older adults, societal stereotypes such as “aging and incompetence,” “social marginalization,” and “isolation” may be internalized as self-stigmatizing feelings, adversely impacting their self-worth. Such stereotypes may act as predictors of future self-stigmatization, influencing older adults’ sense of self-worth and social identity. The long-term perception of ageism may accelerate the formation of stereotypes. Additionally, older adults experiencing higher levels of loneliness and limited social networks often avoid seeking help when faced with negative life events (Jiang and Jiang, 2022). This avoidance may gradually harm their physical and mental health. It is worth noting that in Chinese society, family relationships have traditionally served as a vital source of emotional support and meaning for older adults (Tang et al., 2023). The loss of a spouse often entails not only the absence of intimate companionship but also a weakening of family-based support, especially in the context of increasingly common “empty-nest” households. Such structural changes may further amplify the adverse psychological impact of loneliness. Future interventions should focus on reducing ageism and enhancing social support networks, with particular attention to reinforcing family-based support, which plays a critical role in mitigating loneliness and alleviating anxiety symptoms among widowed older adults.

The findings on the moderating role of cognitive function in mediation models suggest that the interaction between cognitive function and loneliness influences anxiety levels in widowed older adults. Loneliness significantly predicted anxiety across all levels of cognitive impairment. Nonetheless, its predictive effect on anxiety was notably stronger among those with higher levels of cognitive impairment. A previous study suggested that older adults might exert cognitive control over negative biases by comparing the duration for which young and older individuals remembered negative images (Charles et al., 2003). However, for older adults with severe cognitive impairment, such strategies for processing negative emotions may be unlikely to function effectively. Furthermore, social cognitive theory emphasizes how individuals’ perceptions and interpretations of the social environment shape their emotions and behaviors (Bandura, 1991). When older adults experience ageism, cognitive impairment may lead to misinterpretations or negative interpretations of social feedback. For instance, older adults may misperceive support or care from others as rejection or neglect, thereby adversely affecting their mental health. In the present study, we hypothesized that cognitive dysfunction may further increase the risk of anxiety disorders in widowed older adults due to their inability to make effective autonomic adjustments and recognize positive social support when facing loneliness. These findings suggest the need to address cognitive function alongside loneliness when designing psychological interventions.

### 4.1 Advice

Although limited statistical data exists on ageism against older people in China, the scope and impact of ageism may be more pervasive and profound than commonly assumed. Therefore, it is essential to explore strategies for minimizing the occurrence of ageism. A systematic review of studies conducted between 2013 and 2023 has suggested that intergenerational interventions, by fostering understanding and connection between different age groups, may effectively reduce ageist attitudes (Sánchez Sánchez and Montero García, 2025). Furthermore, intergenerational communication may also reduce loneliness among older adults, addressing a critical factor linked to ageism and mental health. A study on intergenerational friendships between older adults and school students in a nursing home found that such interactions expanded older adults’ social networks and reduced their feelings of loneliness (Zamir et al., 2021). Hence, future interventions should prioritize the development and implementation of this method.

In addition to intergenerational strategies, enhancing neighborhood relationships within community settings is also essential for mitigating loneliness among widowed older adults (Zheng and Yan, 2024). In the Chinese context, community-based activities such as playing chess, mahjong, or engaging in traditional group games not only provide entertainment but also serve as important platforms for developing friendships, fostering a sense of belonging, and expanding social support networks. Regular participation in such culturally meaningful activities can reduce feelings of isolation and strengthen connectedness within local communities.

At the same time, family support remains a cornerstone of psychological well-being for older adults in Chinese society, where filial piety and intergenerational caregiving are deeply rooted cultural values (Pang et al., 2023). Adult children can play a pivotal role by maintaining regular emotional contact, providing instrumental assistance, and actively participating in decision-making related to their parents’ care. Community-based programs could also be designed to facilitate and encourage stronger family involvement—such as family counseling sessions and intergenerational bonding activities—so as to reinforce the protective effects of family support on mental health.

Additionally, the role of cognitive function should not be overlooked, and interventions should integrate cognitive training alongside social support. Recent studies indicate that comprehensive approaches combining cognitive stimulation with meaningful social engagement can yield synergistic effects. For instance, Zhu et al. (2022) demonstrated that a multi-domain intervention—incorporating cognitive training, Tai Chi, and group counseling—significantly enhanced cognitive performance and emotional wellbeing among community-dwelling older adults in China. Such holistic strategies acknowledge the intricate interrelationship between cognitive and social dimensions in the aging process, potentially offering more sustainable and effective solutions for mitigating the negative psychological impacts of loneliness.

### 4.2 Limitations

The limitations of this study must be fully acknowledged when interpreting its results. First, the cross-sectional design of the study precludes establishing causal relationships between age discrimination, loneliness, cognitive functioning, and anxiety. Second, the assessment of anxiety relied on retrospective self-reports, which may have been influenced by selective memory and information bias, potentially distorting the findings. Consequently, future studies should employ a longitudinal design to further validate these findings. Moreover, while the current sample size is adequate for this study’s purposes, the exclusive sampling from Shenyang—a single city in China—may limit the generalizability and representativeness of the findings. Future research should incorporate samples from a broader range of regions and more diverse populations to enhance the reliability and applicability of the results to widowed older adults across China. Furthermore, one key contextual variable not included in this study is the duration of widowhood, or the number of years since the loss of a spouse. This factor may meaningfully influence levels of loneliness and anxiety among widowed older adults. Individuals who have been recently widowed are more likely to experience intense psychological distress compared to those who have had more time to adjust to their loss. Future studies should consider including the duration of widowhood as a control variable to better capture individual differences in adaptation processes.

## Data Availability

The raw data supporting the conclusions of this article will be made available by the authors, without undue reservation.
