# Supplementary material for: The relationship between ageism, loneliness, and anxiety in widowed older adults: cognitive function as a moderator in the mediated model
Source: Front Psychol. 2025 Jun 18;16:1624197. doi: 10.3389/fpsyg.2025.1624197 (PMC12213834; doi:10.3389/fpsyg.2025.1624197)
Supplement: Supplementary file 1 [file Supplementary_file_1.docx]

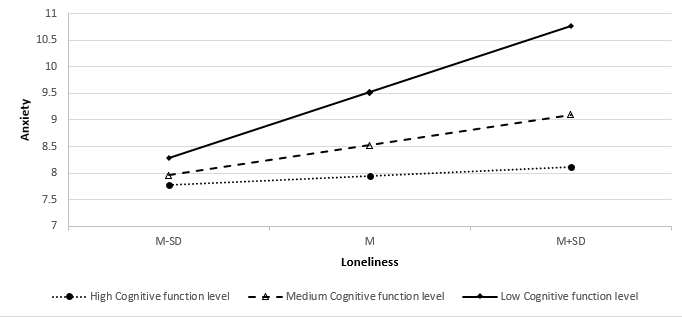


**Figure S1.** Moderating effect of cognitive function levels on the relationship between loneliness and anxiety.
